# 3,4-*O*-Isopropyl­idene-2,7-di-*O*-*p*-tolyl­sulfonyl-α-l-*xylo*-3-heptulo-3,6-furan­osononitrile

**DOI:** 10.1107/S1600536809032565

**Published:** 2009-09-12

**Authors:** Liang Ma, Yun-Feng Li, Xiang-Bao Meng, Zhong-Jun Li

**Affiliations:** a Department of Chemical Biology, School of Pharmaceutical Sciences, Peking University, Beijing 100191, People’s Republic of China; b State Key Laboratory of Natural and Biomimetic Drugs, Peking University, Beijing 100191, People’s Republic of China

## Abstract

In the title compound, C_24_H_27_NO_10_S_2_, derived from l-sorbofuran­ose, the fused five-membered rings display envelope conformations. The two tosyl­ate branches are in equatorial positions with respect to the furan­ose ring, while the hydr­oxy group is in the axial position. In the crystal structure, the hydr­oxy group is involved in inter­molecular O—H⋯O hydrogen bonds, linking mol­ecules in chains along [100].

## Related literature

For details of the synthesis, see: Bianchi *et al.* (2001 ▶); Georges & Fraser-Reid (1984 ▶); Sharma *et al.* (2003 ▶); Szarek *et al.* (1997 ▶).
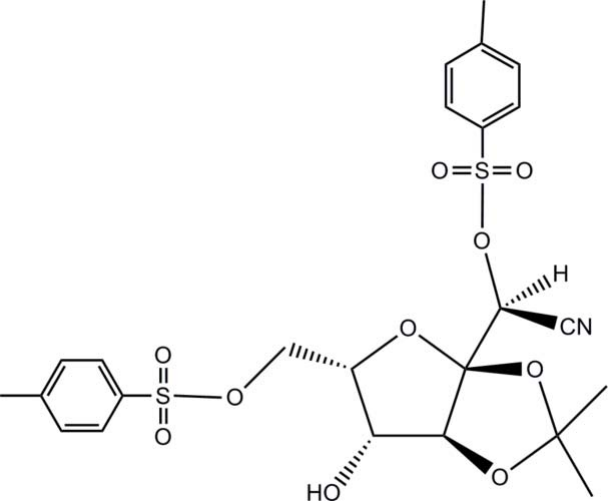


## Experimental

### 

#### Crystal data

C_24_H_27_NO_10_S_2_
*M* *_r_* = 553.59
Monoclinic, 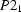

*a* = 5.6342 (11) Å
*b* = 28.771 (6) Å
*c* = 8.3226 (17) Å
β = 103.00 (3)°
*V* = 1314.5 (5) Å^3^
*Z* = 2
Mo *K*α radiation
μ = 0.26 mm^−1^
*T* = 113 K
0.16 × 0.12 × 0.08 mm

#### Data collection

Rigaku Saturn CCD area-detector diffractometer
Absorption correction: multi-scan (*CrystalClear*; Rigaku, 2005 ▶) *T*_min_ = 0.960, *T*_max_ = 0.980
8980 measured reflections
4781 independent reflections
4228 reflections with *I* > 2σ(*I*)
*R*_int_ = 0.039

#### Refinement

*R*[*F* ^2^ > 2σ(*F* ^2^)] = 0.049
*wR*(*F* ^2^) = 0.127
*S* = 1.05
4781 reflections
339 parameters
1 restraint
H-atom parameters constrained
Δρ_max_ = 0.31 e Å^−3^
Δρ_min_ = −0.51 e Å^−3^
Absolute structure: Flack (1983 ▶), 1570 Friedel pairs
Flack parameter: 0.07 (7)

### 

Data collection: *CrystalClear* (Rigaku, 2005 ▶); cell refinement: *CrystalClear*; data reduction: *CrystalClear*; program(s) used to solve structure: *SHELXS97* (Sheldrick, 2008 ▶); program(s) used to refine structure: *SHELXL97* (Sheldrick, 2008 ▶); molecular graphics: *SHELXTL* (Sheldrick, 2008 ▶); software used to prepare material for publication: *SHELXTL*.

## Supplementary Material

Crystal structure: contains datablocks I, global. DOI: 10.1107/S1600536809032565/bh2226sup1.cif

Structure factors: contains datablocks I. DOI: 10.1107/S1600536809032565/bh2226Isup2.hkl

Additional supplementary materials:  crystallographic information; 3D view; checkCIF report

## Figures and Tables

**Table 1:** Hydrogen-bond geometry (Å, °)

*D*—H⋯*A*	*D*—H	H⋯*A*	*D*⋯*A*	*D*—H⋯*A*
O5—H5⋯O4^i^	0.82	2.03	2.844 (3)	169

Symmetry code: (i) 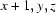.

**Fig. 1.**
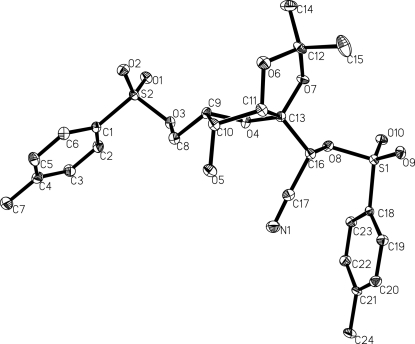
A view of the title molecule, showing the atom-labelling scheme. Displacement ellipsoids are drawn at the 30% probability level. H atoms are represented by circles of arbitrary size.

## supplementary crystallographic information

### Comment

The skeleton of the title compound is a derivative of L-sorbofuranose and consists of two tosyl, a cyano and a bridged ring. Both five-membered rings display an envelope conformation. The hydroxy group lies in axial bond of the furanose ring. The two tosylate branches lie in equatorial bonds of the five-membered furanose ring. The synthesis has been adapted from published procedures (Bianchi *et al.*, 2001; Georges & Fraser-Reid, 1984; Sharma *et al.*, 2003; Szarek *et al.*, 1997).

### Experimental

The title compound was synthesized from the original compound 2,3:4,6-di-*O*-isopropylidene-α-L-sorbofuranose and the procedures are as follows: 2,3:4,6-di-*O*-isopropylidene-α-L-*xylo*-hexos-2-ulofuranose (obtained from the oxidization of 2,3:4,6-di-*O*-isopropylidene-α-L-sorbofuranose) was dissolved in HOAc (80%), followed by addition of NaCN, and after stirred at room temperature for 24 h., 3,4:5,7-di-*O*-isopropylidene-α-L-*xylo*-3-heptulo-3,6-furanosononititrile was formed. The reaction mixture was then directly heated at 323 K for 12 h. to remove 4,6-isopropylidene, yielding 3,4-*O*-isopropylidene-α-L-*xylo*-3-heptulo-3,6-furanosononititrile as a white solid after concentration *in vacuo* and subsequent column chromatography (petroleum-ethyl acetate 1:1). Finally 3,4-*O*-isopropylidene-α-L-*xylo*-3-heptulo-3,6-furanosononititrile was dissolved in pyridine followed by addition of tosyl chloride, and the reaction mixture was stirred at room temperature for 12 h., to obtain the title compound (55% yield over four steps), which was crystallized from petroleum-ethyl acetate (4:1). After a week at room temperature colourless block-like crystals were obtained.

### Refinement

The completeness of diffraction data was limited to 0.945, because the crystal was lost before data collection was completed. The hydroxy H atom H5 was found in a difference map and refined with O—H bond length constrained to 0.82 Å, using a riding approximation, with *U*_iso_(H5) = 1.5*U*_eq_(O5). C-bound H atoms were positioned geometrically (C—H = 0.93–0.98 Å) and refined as riding, with *U*_iso_(H)=1.2–1.5*U*_eq_(C). The expected absolute configuration was confirmed by the refinement of a Flack parameter based on 1570 measured Friedel pairs.

### Crystal data

**Table:**

C_24_H_27_NO_10_S_2_	*F*(000) = 580
*M**_r_* = 553.59	*D*_x_ = 1.399 Mg m^−^^3^
Monoclinic, *P*2_1_	Mo *K*α radiation, λ = 0.71073 Å
Hall symbol: P 2yb	Cell parameters from 3787 reflections
*a* = 5.6342 (11) Å	θ = 1.5–27.5°
*b* = 28.771 (6) Å	µ = 0.26 mm^−^^1^
*c* = 8.3226 (17) Å	*T* = 113 K
β = 103.00 (3)°	Block, colourless
*V* = 1314.5 (5) Å^3^	0.16 × 0.12 × 0.08 mm
*Z* = 2	

### Data collection

**Table:**

Rigaku Saturn CCD area-detector diffractometer	4781 independent reflections
Radiation source: rotating anode	4228 reflections with *I* > 2σ(*I*)
confocal	*R*_int_ = 0.039
Detector resolution: 7.31 pixels mm^-1^	θ_max_ = 27.9°, θ_min_ = 2.5°
ω and φ scans	*h* = −7→7
Absorption correction: multi-scan (*CrystalClear*; Rigaku, 2005)	*k* = −37→36
*T*_min_ = 0.960, *T*_max_ = 0.980	*l* = −8→10
8980 measured reflections	

### Refinement

**Table:**

Refinement on *F*^2^	Secondary atom site location: difference Fourier map
Least-squares matrix: full	Hydrogen site location: inferred from neighbouring sites
*R*[*F*^2^ > 2σ(*F*^2^)] = 0.049	H-atom parameters constrained
*wR*(*F*^2^) = 0.127	*w* = 1/[σ^2^(*F*_o_^2^) + (0.0656*P*)^2^] where *P* = (*F*_o_^2^ + 2*F*_c_^2^)/3
*S* = 1.05	(Δ/σ)_max_ = 0.001
4781 reflections	Δρ_max_ = 0.31 e Å^−^^3^
339 parameters	Δρ_min_ = −0.51 e Å^−^^3^
1 restraint	Absolute structure: Flack (1983), 1570 Friedel pairs
Primary atom site location: structure-invariant direct methods	Flack parameter: 0.07 (7)

### Fractional atomic coordinates and isotropic or equivalent isotropic displacement parameters (Å^2^)

**Table:**

	*x*	*y*	*z*	*U*_iso_*/*U*_eq_	
S1	−0.32882 (13)	0.19945 (2)	0.07345 (11)	0.01793 (18)	
S2	−0.17570 (13)	0.48019 (2)	0.32977 (11)	0.01893 (18)	
N1	0.2652 (6)	0.26412 (11)	0.3805 (5)	0.0315 (7)	
O1	−0.4314 (4)	0.48295 (9)	0.3207 (3)	0.0262 (6)	
O2	−0.0775 (4)	0.49967 (8)	0.2009 (3)	0.0252 (6)	
O3	−0.1224 (4)	0.42629 (8)	0.3418 (3)	0.0210 (5)	
O4	−0.0948 (4)	0.34836 (7)	0.1318 (3)	0.0196 (5)	
O5	0.4380 (4)	0.35399 (8)	0.2068 (3)	0.0244 (5)	
H5	0.5668	0.3487	0.1801	0.037*	
O6	0.0421 (4)	0.35979 (9)	−0.2024 (3)	0.0267 (6)	
O7	−0.2485 (4)	0.31572 (8)	−0.1269 (3)	0.0206 (5)	
O8	−0.2598 (4)	0.25334 (7)	0.1007 (3)	0.0196 (5)	
O9	−0.2258 (4)	0.18218 (8)	−0.0567 (3)	0.0232 (5)	
O10	−0.5865 (4)	0.19956 (9)	0.0566 (3)	0.0232 (5)	
C1	−0.0181 (6)	0.50230 (11)	0.5188 (5)	0.0199 (7)	
C2	−0.1140 (7)	0.49476 (12)	0.6580 (5)	0.0283 (9)	
H2	−0.2577	0.4781	0.6489	0.034*	
C3	0.0070 (7)	0.51242 (13)	0.8098 (5)	0.0313 (9)	
H3	−0.0570	0.5076	0.9021	0.038*	
C4	0.2236 (7)	0.53735 (12)	0.8252 (6)	0.0300 (9)	
C5	0.3154 (6)	0.54421 (13)	0.6857 (6)	0.0309 (9)	
H5A	0.4591	0.5609	0.6944	0.037*	
C6	0.1973 (6)	0.52669 (12)	0.5336 (5)	0.0245 (8)	
H6	0.2627	0.5313	0.4417	0.029*	
C7	0.3506 (8)	0.55689 (15)	0.9890 (6)	0.0409 (11)	
H7A	0.5230	0.5576	0.9960	0.061*	
H7B	0.3171	0.5377	1.0757	0.061*	
H7C	0.2932	0.5879	1.0000	0.061*	
C8	0.1045 (5)	0.40951 (11)	0.3035 (5)	0.0219 (8)	
H8A	0.1761	0.3855	0.3814	0.026*	
H8B	0.2201	0.4349	0.3119	0.026*	
C9	0.0493 (5)	0.39035 (10)	0.1331 (5)	0.0188 (7)	
H9	−0.0421	0.4132	0.0560	0.023*	
C10	0.2720 (5)	0.37409 (11)	0.0732 (5)	0.0201 (7)	
H10	0.3464	0.3996	0.0237	0.024*	
C11	0.1613 (5)	0.33768 (11)	−0.0543 (4)	0.0203 (7)	
H11	0.2793	0.3142	−0.0711	0.024*	
C12	−0.1785 (6)	0.33503 (13)	−0.2688 (5)	0.0234 (7)	
C13	−0.0521 (5)	0.31694 (10)	0.0094 (5)	0.0176 (7)	
C14	−0.3723 (7)	0.36825 (15)	−0.3504 (5)	0.0363 (10)	
H14A	−0.3906	0.3922	−0.2737	0.054*	
H14B	−0.5236	0.3519	−0.3856	0.054*	
H14C	−0.3271	0.3820	−0.4443	0.054*	
C15	−0.1348 (8)	0.29602 (17)	−0.3803 (6)	0.0454 (12)	
H15A	−0.0884	0.3088	−0.4753	0.068*	
H15B	−0.2813	0.2782	−0.4149	0.068*	
H15C	−0.0068	0.2763	−0.3216	0.068*	
C16	−0.0202 (5)	0.26825 (11)	0.0850 (4)	0.0177 (7)	
H16	0.0402	0.2471	0.0109	0.021*	
C17	0.1446 (6)	0.26673 (11)	0.2511 (5)	0.0227 (7)	
C18	−0.1844 (5)	0.17379 (10)	0.2592 (4)	0.0159 (6)	
C19	0.0301 (6)	0.14921 (11)	0.2676 (5)	0.0222 (7)	
H19	0.0925	0.1446	0.1745	0.027*	
C20	0.1489 (6)	0.13163 (11)	0.4215 (5)	0.0252 (8)	
H20	0.2918	0.1147	0.4297	0.030*	
C21	0.0598 (6)	0.13870 (11)	0.5629 (5)	0.0240 (8)	
C22	−0.1557 (6)	0.16375 (12)	0.5487 (5)	0.0258 (8)	
H22	−0.2179	0.1687	0.6418	0.031*	
C23	−0.2790 (6)	0.18142 (11)	0.3976 (5)	0.0212 (7)	
H23	−0.4226	0.1981	0.3891	0.025*	
C24	0.1933 (8)	0.12001 (14)	0.7275 (5)	0.0345 (10)	
H24A	0.3418	0.1054	0.7159	0.052*	
H24B	0.0928	0.0976	0.7662	0.052*	
H24C	0.2304	0.1451	0.8052	0.052*	

### Atomic displacement parameters (Å^2^)

**Table:**

	*U*^11^	*U*^22^	*U*^33^	*U*^12^	*U*^13^	*U*^23^
S1	0.0172 (3)	0.0193 (3)	0.0174 (5)	−0.0009 (3)	0.0043 (3)	−0.0008 (3)
S2	0.0184 (4)	0.0219 (4)	0.0171 (5)	−0.0002 (3)	0.0051 (3)	−0.0031 (3)
N1	0.0297 (15)	0.0327 (16)	0.030 (2)	0.0054 (13)	0.0026 (15)	0.0022 (15)
O1	0.0184 (10)	0.0325 (13)	0.0280 (16)	0.0019 (10)	0.0060 (11)	−0.0039 (12)
O2	0.0333 (13)	0.0257 (11)	0.0195 (15)	0.0003 (10)	0.0122 (12)	0.0025 (11)
O3	0.0190 (10)	0.0209 (11)	0.0250 (15)	−0.0015 (9)	0.0092 (10)	−0.0048 (10)
O4	0.0154 (10)	0.0175 (10)	0.0292 (15)	−0.0021 (8)	0.0120 (10)	−0.0044 (10)
O5	0.0133 (10)	0.0336 (13)	0.0263 (15)	0.0013 (9)	0.0044 (10)	−0.0012 (11)
O6	0.0279 (12)	0.0332 (12)	0.0208 (15)	−0.0061 (10)	0.0095 (12)	0.0024 (11)
O7	0.0162 (10)	0.0277 (12)	0.0160 (14)	−0.0020 (9)	−0.0004 (10)	0.0049 (10)
O8	0.0144 (9)	0.0201 (10)	0.0253 (15)	0.0019 (9)	0.0063 (10)	0.0009 (10)
O9	0.0295 (12)	0.0239 (11)	0.0186 (14)	−0.0007 (10)	0.0107 (11)	−0.0050 (10)
O10	0.0142 (9)	0.0299 (11)	0.0238 (15)	−0.0014 (9)	0.0009 (10)	0.0019 (12)
C1	0.0204 (14)	0.0194 (14)	0.021 (2)	0.0003 (12)	0.0069 (15)	−0.0031 (14)
C2	0.0321 (18)	0.0293 (17)	0.025 (2)	−0.0092 (14)	0.0108 (18)	0.0008 (16)
C3	0.041 (2)	0.0345 (19)	0.020 (2)	−0.0061 (16)	0.0082 (18)	0.0003 (16)
C4	0.036 (2)	0.0228 (16)	0.031 (3)	0.0041 (14)	0.0057 (18)	−0.0008 (16)
C5	0.0225 (17)	0.0346 (19)	0.035 (3)	−0.0055 (15)	0.0051 (17)	−0.0051 (18)
C6	0.0235 (16)	0.0269 (16)	0.026 (2)	−0.0009 (13)	0.0120 (16)	−0.0033 (15)
C7	0.050 (2)	0.036 (2)	0.032 (3)	0.0001 (18)	−0.001 (2)	−0.005 (2)
C8	0.0146 (13)	0.0243 (16)	0.028 (2)	0.0017 (12)	0.0065 (14)	−0.0043 (15)
C9	0.0149 (13)	0.0161 (13)	0.026 (2)	−0.0016 (11)	0.0054 (14)	−0.0006 (13)
C10	0.0150 (13)	0.0256 (15)	0.020 (2)	−0.0014 (12)	0.0055 (14)	−0.0026 (14)
C11	0.0156 (14)	0.0270 (15)	0.020 (2)	−0.0012 (12)	0.0070 (14)	−0.0046 (14)
C12	0.0213 (15)	0.0362 (18)	0.0130 (19)	−0.0070 (14)	0.0048 (14)	−0.0006 (15)
C13	0.0138 (13)	0.0168 (14)	0.022 (2)	0.0024 (11)	0.0030 (13)	−0.0015 (14)
C14	0.0342 (19)	0.043 (2)	0.029 (3)	−0.0029 (17)	0.0004 (19)	0.0153 (19)
C15	0.039 (2)	0.062 (3)	0.040 (3)	−0.011 (2)	0.018 (2)	−0.026 (2)
C16	0.0134 (13)	0.0208 (14)	0.0194 (19)	−0.0005 (11)	0.0047 (13)	−0.0009 (13)
C17	0.0215 (15)	0.0241 (15)	0.024 (2)	0.0028 (13)	0.0083 (16)	0.0011 (14)
C18	0.0181 (14)	0.0180 (14)	0.0123 (18)	−0.0009 (11)	0.0049 (14)	−0.0020 (12)
C19	0.0217 (15)	0.0247 (15)	0.021 (2)	0.0018 (13)	0.0055 (15)	0.0009 (14)
C20	0.0202 (15)	0.0239 (16)	0.030 (2)	0.0010 (13)	0.0032 (16)	0.0024 (15)
C21	0.0273 (17)	0.0215 (15)	0.018 (2)	−0.0019 (13)	−0.0061 (16)	0.0020 (14)
C22	0.0336 (19)	0.0255 (16)	0.020 (2)	0.0025 (14)	0.0090 (17)	0.0019 (15)
C23	0.0256 (16)	0.0247 (15)	0.0153 (19)	0.0011 (13)	0.0087 (15)	0.0014 (14)
C24	0.041 (2)	0.0337 (19)	0.021 (2)	−0.0005 (16)	−0.0092 (19)	0.0051 (18)

### Geometric parameters (Å, °)

**Table:**

S1—O10	1.427 (2)	C8—H8A	0.9700
S1—O9	1.428 (2)	C8—H8B	0.9700
S1—O8	1.602 (2)	C9—C10	1.524 (4)
S1—C18	1.741 (4)	C9—H9	0.9800
S2—O2	1.427 (3)	C10—C11	1.521 (4)
S2—O1	1.428 (2)	C10—H10	0.9800
S2—O3	1.578 (2)	C11—C13	1.539 (4)
S2—C1	1.743 (4)	C11—H11	0.9800
N1—C17	1.139 (5)	C12—C14	1.494 (5)
O3—C8	1.467 (3)	C12—C15	1.512 (5)
O4—C13	1.423 (4)	C13—C16	1.530 (4)
O4—C9	1.454 (3)	C14—H14A	0.9600
O5—C10	1.406 (4)	C14—H14B	0.9600
O5—H5	0.8200	C14—H14C	0.9600
O6—C11	1.415 (4)	C15—H15A	0.9600
O6—C12	1.431 (4)	C15—H15B	0.9600
O7—C13	1.396 (4)	C15—H15C	0.9600
O7—C12	1.438 (4)	C16—C17	1.483 (5)
O8—C16	1.450 (3)	C16—H16	0.9800
C1—C6	1.383 (4)	C18—C19	1.388 (4)
C1—C2	1.401 (5)	C18—C23	1.391 (5)
C2—C3	1.390 (6)	C19—C20	1.399 (5)
C2—H2	0.9300	C19—H19	0.9300
C3—C4	1.396 (5)	C20—C21	1.394 (5)
C3—H3	0.9300	C20—H20	0.9300
C4—C5	1.388 (6)	C21—C22	1.394 (5)
C4—C7	1.499 (6)	C21—C24	1.506 (5)
C5—C6	1.386 (6)	C22—C23	1.389 (5)
C5—H5A	0.9300	C22—H22	0.9300
C6—H6	0.9300	C23—H23	0.9300
C7—H7A	0.9600	C24—H24A	0.9600
C7—H7B	0.9600	C24—H24B	0.9600
C7—H7C	0.9600	C24—H24C	0.9600
C8—C9	1.488 (5)		
			
O10—S1—O9	120.29 (15)	O6—C11—C13	102.7 (2)
O10—S1—O8	102.76 (13)	C10—C11—C13	104.9 (3)
O9—S1—O8	108.47 (13)	O6—C11—H11	112.9
O10—S1—C18	110.50 (14)	C10—C11—H11	112.9
O9—S1—C18	109.72 (15)	C13—C11—H11	112.9
O8—S1—C18	103.57 (14)	O6—C12—O7	104.5 (3)
O2—S2—O1	119.71 (16)	O6—C12—C14	109.8 (3)
O2—S2—O3	109.48 (13)	O7—C12—C14	108.2 (3)
O1—S2—O3	103.49 (13)	O6—C12—C15	111.1 (3)
O2—S2—C1	109.25 (16)	O7—C12—C15	109.3 (3)
O1—S2—C1	109.28 (16)	C14—C12—C15	113.5 (4)
O3—S2—C1	104.48 (15)	O7—C13—O4	111.5 (2)
C8—O3—S2	118.17 (19)	O7—C13—C16	108.0 (2)
C13—O4—C9	110.4 (2)	O4—C13—C16	108.1 (3)
C10—O5—H5	109.5	O7—C13—C11	105.6 (3)
C11—O6—C12	108.3 (3)	O4—C13—C11	105.7 (2)
C13—O7—C12	110.1 (2)	C16—C13—C11	117.9 (2)
C16—O8—S1	118.35 (17)	C12—C14—H14A	109.5
C6—C1—C2	119.9 (4)	C12—C14—H14B	109.5
C6—C1—S2	121.6 (3)	H14A—C14—H14B	109.5
C2—C1—S2	118.5 (3)	C12—C14—H14C	109.5
C3—C2—C1	119.6 (3)	H14A—C14—H14C	109.5
C3—C2—H2	120.2	H14B—C14—H14C	109.5
C1—C2—H2	120.2	C12—C15—H15A	109.5
C2—C3—C4	120.8 (4)	C12—C15—H15B	109.5
C2—C3—H3	119.6	H15A—C15—H15B	109.5
C4—C3—H3	119.6	C12—C15—H15C	109.5
C5—C4—C3	118.6 (4)	H15A—C15—H15C	109.5
C5—C4—C7	121.1 (4)	H15B—C15—H15C	109.5
C3—C4—C7	120.4 (4)	O8—C16—C17	107.9 (3)
C6—C5—C4	121.3 (3)	O8—C16—C13	106.4 (2)
C6—C5—H5A	119.3	C17—C16—C13	113.7 (3)
C4—C5—H5A	119.3	O8—C16—H16	109.6
C1—C6—C5	119.8 (3)	C17—C16—H16	109.6
C1—C6—H6	120.1	C13—C16—H16	109.6
C5—C6—H6	120.1	N1—C17—C16	177.1 (4)
C4—C7—H7A	109.5	C19—C18—C23	122.0 (3)
C4—C7—H7B	109.5	C19—C18—S1	119.2 (3)
H7A—C7—H7B	109.5	C23—C18—S1	118.6 (2)
C4—C7—H7C	109.5	C18—C19—C20	117.5 (3)
H7A—C7—H7C	109.5	C18—C19—H19	121.2
H7B—C7—H7C	109.5	C20—C19—H19	121.2
O3—C8—C9	108.9 (3)	C21—C20—C19	122.1 (3)
O3—C8—H8A	109.9	C21—C20—H20	119.0
C9—C8—H8A	109.9	C19—C20—H20	119.0
O3—C8—H8B	109.9	C22—C21—C20	118.4 (3)
C9—C8—H8B	109.9	C22—C21—C24	120.6 (4)
H8A—C8—H8B	108.3	C20—C21—C24	121.0 (3)
O4—C9—C8	108.1 (3)	C23—C22—C21	120.9 (4)
O4—C9—C10	104.0 (2)	C23—C22—H22	119.5
C8—C9—C10	114.5 (3)	C21—C22—H22	119.5
O4—C9—H9	110.0	C22—C23—C18	119.0 (3)
C8—C9—H9	110.0	C22—C23—H23	120.5
C10—C9—H9	110.0	C18—C23—H23	120.5
O5—C10—C11	111.3 (3)	C21—C24—H24A	109.5
O5—C10—C9	108.5 (3)	C21—C24—H24B	109.5
C11—C10—C9	101.6 (2)	H24A—C24—H24B	109.5
O5—C10—H10	111.6	C21—C24—H24C	109.5
C11—C10—H10	111.6	H24A—C24—H24C	109.5
C9—C10—H10	111.6	H24B—C24—H24C	109.5
O6—C11—C10	109.7 (3)		
			
O2—S2—O3—C8	33.2 (3)	C13—O7—C12—O6	−16.2 (3)
O1—S2—O3—C8	161.9 (3)	C13—O7—C12—C14	−133.2 (3)
C1—S2—O3—C8	−83.7 (3)	C13—O7—C12—C15	102.7 (3)
O10—S1—O8—C16	−166.0 (2)	C12—O7—C13—O4	112.8 (3)
O9—S1—O8—C16	−37.6 (3)	C12—O7—C13—C16	−128.6 (3)
C18—S1—O8—C16	78.9 (3)	C12—O7—C13—C11	−1.6 (3)
O2—S2—C1—C6	−12.2 (3)	C9—O4—C13—O7	−105.8 (3)
O1—S2—C1—C6	−144.9 (3)	C9—O4—C13—C16	135.5 (3)
O3—S2—C1—C6	104.8 (3)	C9—O4—C13—C11	8.4 (3)
O2—S2—C1—C2	168.0 (3)	O6—C11—C13—O7	18.8 (3)
O1—S2—C1—C2	35.3 (3)	C10—C11—C13—O7	133.5 (3)
O3—S2—C1—C2	−75.0 (3)	O6—C11—C13—O4	−99.5 (3)
C6—C1—C2—C3	0.8 (5)	C10—C11—C13—O4	15.2 (3)
S2—C1—C2—C3	−179.4 (3)	O6—C11—C13—C16	139.6 (3)
C1—C2—C3—C4	−0.3 (6)	C10—C11—C13—C16	−105.7 (3)
C2—C3—C4—C5	0.1 (6)	S1—O8—C16—C17	−92.6 (3)
C2—C3—C4—C7	179.0 (4)	S1—O8—C16—C13	145.0 (2)
C3—C4—C5—C6	−0.3 (6)	O7—C13—C16—O8	−48.4 (3)
C7—C4—C5—C6	−179.2 (4)	O4—C13—C16—O8	72.5 (3)
C2—C1—C6—C5	−1.0 (5)	C11—C13—C16—O8	−167.8 (3)
S2—C1—C6—C5	179.2 (3)	O7—C13—C16—C17	−167.0 (3)
C4—C5—C6—C1	0.8 (6)	O4—C13—C16—C17	−46.2 (3)
S2—O3—C8—C9	−100.7 (3)	C11—C13—C16—C17	73.5 (4)
C13—O4—C9—C8	−150.7 (2)	O10—S1—C18—C19	147.6 (3)
C13—O4—C9—C10	−28.7 (3)	O9—S1—C18—C19	12.7 (3)
O3—C8—C9—O4	−67.9 (3)	O8—S1—C18—C19	−103.0 (3)
O3—C8—C9—C10	176.7 (3)	O10—S1—C18—C23	−36.9 (3)
O4—C9—C10—O5	−81.1 (3)	O9—S1—C18—C23	−171.8 (2)
C8—C9—C10—O5	36.6 (4)	O8—S1—C18—C23	72.5 (3)
O4—C9—C10—C11	36.3 (3)	C23—C18—C19—C20	0.7 (5)
C8—C9—C10—C11	154.0 (3)	S1—C18—C19—C20	176.0 (2)
C12—O6—C11—C10	−140.6 (3)	C18—C19—C20—C21	−0.9 (5)
C12—O6—C11—C13	−29.4 (3)	C19—C20—C21—C22	0.6 (5)
O5—C10—C11—O6	−166.2 (2)	C19—C20—C21—C24	−179.1 (3)
C9—C10—C11—O6	78.4 (3)	C20—C21—C22—C23	−0.2 (5)
O5—C10—C11—C13	84.1 (3)	C24—C21—C22—C23	179.5 (3)
C9—C10—C11—C13	−31.3 (3)	C21—C22—C23—C18	0.0 (5)
C11—O6—C12—O7	29.1 (3)	C19—C18—C23—C22	−0.3 (5)
C11—O6—C12—C14	145.0 (3)	S1—C18—C23—C22	−175.7 (3)
C11—O6—C12—C15	−88.6 (4)		

### Hydrogen-bond geometry (Å, °)

**Table:**

*D*—H···*A*	*D*—H	H···*A*	*D*···*A*	*D*—H···*A*
O5—H5···O4^i^	0.82	2.03	2.844 (3)	169

Symmetry codes: (i) *x*+1, *y*, *z*.
